# RNA-seq and *In Vitro* Experiments Reveal the Protective Effect of Curcumin against 5-Fluorouracil-Induced Intestinal Mucositis via IL-6/STAT3 Signaling Pathway

**DOI:** 10.1155/2021/8286189

**Published:** 2021-07-21

**Authors:** Xuan-ying Wang, Bo Zhang, Yi Lu, Lu Xu, Yi-jie Wang, Bi-yu Cai, Qing-hua Yao

**Affiliations:** ^1^Second Clinical Medical College, Zhejiang Chinese Medical University, Hangzhou, Zhejiang Province 310053, China; ^2^Department of Integrated Traditional Chinese and Western Medicine, The Cancer Hospital of the University of Chinese Academy of Sciences (Zhejiang Cancer Hospital), Institute of Basic Medicine and Cancer (IBMC), Chinese Academy of Sciences, Hangzhou, Zhejiang Province 310022, China; ^3^Department of Clinical Nutrition, The Cancer Hospital of the University of Chinese Academy of Sciences (Zhejiang Cancer Hospital), Institute of Basic Medicine and Cancer (IBMC), Chinese Academy of Sciences, Hangzhou, Zhejiang Province 310022, China

## Abstract

Although first-line chemotherapy drugs, including 5-fluorouracil (5-FU), remain one of the major choice for cancer treatment, the clinical use is also accompanied with dose-depending toxicities, such as intestinal mucositis (IM), in cancer patients undergoing treatment. IM-induced gastrointestinal adverse reactions become frequent reason to postpone chemotherapy and have negative impacts on therapeutic outcomes and prognosis. Various studies have evidenced the anticancer role of curcumin in many cancers; except for this effect, studies also indicated a protective role of curcumin in intestinal diseases. Therefore, in this study, we investigated the effect of curcumin on inflammation, intestinal epithelial cell damage in an IM model. 5-FU was used to induce the model of IM in intestinal epithelial cells, and curcumin at different concentrations was administrated. The results showed that curcumin efficiently attenuated 5-FU-induced damage to IEC-6 cells, inhibited the levels of inflammatory cytokines, attenuated the 5-FU-induced inhibition on cell viability, and displayed antiapoptosis effect on IEC-6 cells. Further RNA-sequencing analysis and experiment validation found that curcumin displays its protective effect against 5-FU-induced IM in intestinal epithelial cells by the inhibition of IL-6/STAT3 signaling pathway. Taken together, these findings suggested that curcumin may be provided as a therapeutic agent in prevention and treatment of chemotherapy-induced IM.

## 1. Introduction

Chemotherapy remains one of the major strategies for cancer treatment, with the ability of inhibiting cancer cell growth and metastasis to a certain extent. But there are also some unavoidable problems occurring during the treatment [1]. Intestinal mucositis (IM) is a major adverse effect induced by chemotherapeutic agents, such as 5-fluorouracil (5-FU), in cancer patients undergoing treatment [2]. 5-FU is a commonest first-line systemic chemotherapy drug used to fight against numerous types of cancers, particularly colorectal cancer [3–5]. But the use of 5-FU in colorectal cancer patients is often accompanied by alimentary system injury through the activation of inflammation response and toxicity to intestinal mucosa and intestinal epithelial cell, thus resulting in IM [6, 7]. A series of gastrointestinal reactions, including vomiting, diarrhea, indigestion, ulceration, and loss of appetite, are the main clinical manifestations of IM [8]. Approximately 5 ~ 15% of the patients suffered from IM worldwide, which is a frequent reason to postpone chemotherapy and a cause of mortality in chemotherapy patients [2]. Current management options for IM mainly focused on supportive care. Thus, curative or preventative medicines are unmet in order to reduce or eliminate the severity of IM symptoms induced by chemotherapy.

Chemotherapy-induced IM is mainly characterized by destruction of the intestinal mucosal barrier. The intestinal epithelial cell is a critical part to maintain the homeostasis of the intestinal mucosal barrier. 5-FU acts anticancer effect by its cytotoxicity, inhibiting DNA synthesis of not only tumor cells but also normal cells, especially the sensitive intestinal epithelial cell of the intestinal mucosal barrier [9]. In addition, 5-FU activates a diverse range of proinflammatory pathways to excessive release proinflammatory cytokines, such as TNF-*α* and IL-6, culminating in histopathological damages in the intestinal mucosa [10]. Therefore, inflammation is a key event in IM, and many studies also focus on preventing 5-FU-induced IM through the regulation of inflammation and related signals.

Curcumin is a well-known dietary polyphenol found from *Curcuma longa L.* (turmeric), a perennial herb belonging to the family Zingiberaceae. It has been used as a traditional medicine for over 2,000 years in China and India [11]. Abundant studies have indicated the beneficial role of curcumin against various chronic diseases, including various types of cancers, diabetes, cardiovascular, pulmonary, neurological, and autoimmune diseases [12]. Particularly, the ability of curcumin to target multiple pathways makes its effect on cancer have been studied mostly [13]. Except for its anticancer effect, studies also indicated a protective role of curcumin in alimentary system. Curcumin can protect human intestinal epithelial cell from inflammatory damage and cell apoptosis induced by IFN-*γ*, indicated the preventive role of curcumin in intestinal damage [14]. Zhang et al. also report that curcumin displays a protective role in intestinal inflamed rat, and this effect is realized by inhibiting cell apoptosis and JAK/STAT pathways [15].

However, up to date, reports about the protective effects and under mechanism of curcumin against 5-FU-induced IM are less known. Therefore, in this study, we investigated the effect of curcumin on intestinal epithelial cell damage and inflammation induced by 5-FU. The discovered protective properties of curcumin in this study may provide a possible option in prevention and treatment 5-FU-induced IM.

## 2. Material and Methods

### 2.1. Cell Culture

The rat intestinal epithelial cell line IEC-6 was obtained from the iCell Bioscience Inc. (Shanghai, China). Cells were cultured in Dulbecco's modified eagle media (DMEM) supplemented with 10% fetal bovine serum (FBS), 100 U/mL penicillin, and 100 *μ*g/mL streptomycin. Cells were maintained at 37°C in a humidified incubator with 5% CO2. The culture medium was changed twice a week.

### 2.2. Plasmid Construction and Cell Transfection

STAT3 overexpressed plasmid and IL-6 overexpressed plasmid were obtained from Shanghai Genchem Co., LTD (China). IEC-6 cells were transfected with these plasmids following the manufacturer's instructions of Lipofectamine 2000 (Invitrogen, USA), and pcDNA vector was transfected as matched negative control (NC).

### 2.3. Cell Treatment

IEC-6 cells were divided into the following groups in this study: 5-FU group: IEC-6 cells were incubated with 5-FU at a concentration of 50 mg/mL for 24 h; 5-FU + curcumin group: cells were incubated with 5-FU at a concentration of 50 mg/mL for 24 h and then further incubated with curcumin at different concentrations (5, 10, and 20 *μ*mol/L); 5-FU + curcumin + STAT3 group: STAT3 overexpressed plasmid transfected IEC-6 cells were incubated with 5-FU for 24 h, with curcumin at 10 *μ*mol/L for another 24 h; and 5-FU + curcumin + IL-6 group: IEC-6 cells firstly transfected with IL-6 overexpressed plasmid, and then, cells were incubated with 5-FU and curcumin at 10 *μ*mol/L for another 24 h. Control group IEC-6 cells were incubated with PBS instead.

### 2.4. CCK-8 Cell Viability Assessment

Briefly, IEC-6 cells were seeded into 96 plates and allowed to adhere for 24 h. Then, the medium was substituted with 5-FU diluted in DMEM or a new medium as control alone for 24 h. Afterwards, curcumin at different concentrations was added into the medium and coincubated with IEC-6 cells for another 24 or 48 h. Subsequently, CCK-8 solution (10 *μ*L) was added into each well, and the optical density (OD) value was then measured using a microplate reader (Molecular Devices, USA).

### 2.5. Colony Formation Assay

After adhesion, IEC-6 cells were treated with 5-FU plus curcumin; cells were trypsinised and plated into 6 well plates for routinely incubation for 14 days. After that, the colonies were fixed with 4% paraformaldehyde and further stained with 0.1% crystal violet. Then, the colonies in each group were counted.

### 2.6. Flow Cytometry Assay

The Annexin-V-FITC/Propidium Iodide (PI) Apoptosis Detection Kit (CoWin Biosciences, China) was used to detect the cell apoptosis. Treated IEC-6 cells were seeded into 6 well plates. The cells were allowed to adhere and washed by PBS twice. Then, they were collected at a density of 1 × 10^6^ cells per well and ringed with 500 *μ*L of binding buffer. After removed, the supernatant and resuspended in binding buffer (200 *μ*L), 5 *μ*L Annexin V-FITC, and 10 *μ*L PI solution were added into the plates and incubated for 15 min in the dark. Followed by the readdition of binding buffer (300 *μ*L), the cell apoptosis was assessed by a flow cytometer (Beckman coulter, USA).

### 2.7. TUNEL Staining Assay

TUNEL staining assay was performed to stain apoptotic cells and detect cell apoptosis. The staining of the treated IEC-6 cells was performed using a TUNEL cell death detection kit (Sangon Biotech, China) according to the manufacturer's instructions. After staining and washing with PBS, cell nuclei were further stained with DAPI solution. The TUNEL positive cells were photographed using an inverted fluorescence microscope (Nikon, Japan), and the TUNEL positive cells were analyzed.

### 2.8. Wound Healing for Cellular Migration

Migration ability of IEC-6 cells was analyzed using wound healing assay. The IEC-6 cells were seeded in a 6-well plate, allowed to adherent, and then treated with the indicated regent. A pipette tip was allowed to draw a line along the ruler at the bottom of the plate. Then, cells were allowed to recover in next 24 h under routine incubation. Pictures of the scratches were photographed at 0 h and 24 h, and the cell migration distance was also detected.

### 2.9. qRT-PCR

Total RNA was isolated according to the instructions of Trizol regent. And the isolated RNA was reversely transcribed into cDNA using a cDNA synthesis kit (CoWin Biosciences, China). The RT-PCR procedure was performed using a SYBR Green qPCR kit (CoWin Biosciences, China) according to the manufacturer's instructions. And the PCR reaction condition was 95°C for 10 min, 95°C for 15 s, and 60°C for 60 s for 40 cycles. The primer sequences of the detected genes were as follows: IL-6, forward primer: 5′-CCAGTTGCCTGGGACT-3′, reverse primer: 5′-TGCCATTGCAACTTTTC-3′; STAT3, forward primer: 5′-GAGCTTGGGGTTCCGACG-3′, reverse primer: 5′-AGGGGTGACCACTGTCTCT-3′; and GAPDH, forward primer: 5′-ATGATTCTACCCACGGCAAG-3′, reverse primer: 5′-CTGGAAGATGGTGATGGGTT-3′.

GAPDH was used as the internal reference, and the mRNA levels was calculated using the 2^-△△Ct^ method.

### 2.10. Western Blotting

The total protein of each group cells was extracted with radio-immunoprecipitation assay (RIPA) lysis buffer, and the extracted protein concentration was detected using a Bradford protein assay (BCA) method (Solarbio, China). Then, the protein samples were separated on 10% sodium dodecyl sulfate-polyacrylamide gel electrophoresis (SDS-PAGE) gels and transferred onto the membranes of nitrocellulose. It was blocked with 5% skim milk at room temperature for 2 h, and then, the membranes were incubated with primary antibody anti-IL-6, anti-Phospho-Stat3, anti-E-cadherin, anti-vimentin, anti-N-cadherin, and anti-*β*-actin (CST, USA) at 4°C overnight. Subsequently, the membranes were further incubated with the secondary antibody (HRP-conjugated) for 2 h at room temperature. The blots were visualised using an enhanced chemiluminescence (ECL) kit and were photographed. The blot density was quantified, and the densitometry was analyzed using the ImageJ software.

### 2.11. RNA-Sequencing

The IEC-6 cells from the control, 5-FU, and 5-FU + curcumin (10 *μ*mol/L) groups were harvested for RNA-sequencing analysis. Generally, total RNA from the cells was isolated and purified using TRIzol reagent (Invitrogen, USA). The RNA purity was quantified using NanoDrop ND-1000 (NanoDrop, USA); the RNA integrity was assessed by Bioanalyzer 2100 (Agilent, USA) and confirmed by denaturing agarose gel electrophoresis. Poly (A) RNA was purified from 1 *μ*g total RNA using Dynabeads Oligo (dT)25-61005 (Thermo Fisher, USA). Then, the obtained poly (A) RNA was fragmented into small pieces using Magnesium RNA Fragmentation Module (NEB, cat. e6150, USA) under 94°C for 5-7 min. Then, the RNA fragments were reverse-transcribed for cDNA library preparation and RNA-sequencing following the manufacturer's protocol. The 2 × 150 bp paired-end sequencing (PE150) was performed on an Illumina Novaseq™ 6000 (LC-Bio Technology CO., Ltd., China) following the vendor's recommended protocol.

### 2.12. RNA-Sequencing Data Analysis

The raw reads that contained adaptor contamination were firstly removed using the Cutadapt software (https://cutadapt.readthedocs.io/en/stable/,version:cutadapt-1.9).Then, the HISAT2 software (https://daehwankimlab.github.io/hisat2/,version:hisat2-2.0.4) was used to map reads to the genome, and the mapped reads of each sample were assembled using StringTie (http://ccb.jhu.edu/software/stringtie/,version:stringtie-1.3.4d.Linux_x86_64) with default parameters. After that, transcriptomes from all the samples were merged to reconstruct a comprehensive transcriptome using the gffcompare software (http://ccb.jhu.edu/software/stringtie/gffcompare.shtml,version:gffcompare-0.9.8.Linux_x86_64). After the final transcriptome was generated, StringTie and ballgown (http://www.bioconductor.org/packages/release/bioc/html/ballgown.html) were used to estimate the expression levels of all transcripts and calculate FPKM for mRNAs. The thresholds of differentially expressed genes (DEGs) were ∣log fold change (FC) | >1 and *p* value < 0.05 analyzed by R package edgeR (https://bioconductor.org/packages/release/bioc/html/edgeR.html) or DESeq2 (http://www.bioconductor.org/packages/release/bioc/html/DESeq2.html). The analysis of protein-protein intersection (PPI), Gene Ontology (GO) enrichment, and Kyoto Encyclopedia of Genes and Genomes (KEGG) enrichment of the differentially expressed mRNAs was also performed using online tool STRING (https://string.embl.de/) and R package.

### 2.13. Statistical Analysis

All *in vitro* experiments were repeated 3 times. All data were expressed as mean ± standard error mean (SEM). The statistical analysis was performed using the SPSS software. Differences between unpaired comparisons between two groups were analyzed using the Student's *t*-test. One-way ANOVA followed by Student Newman-Keuls was used for multiple group comparisons.

## 3. Results

### 3.1. Effect of Curcumin on the Viability of the 5-FU-Stimulated IEC-6 Cells

**Figure 1:**
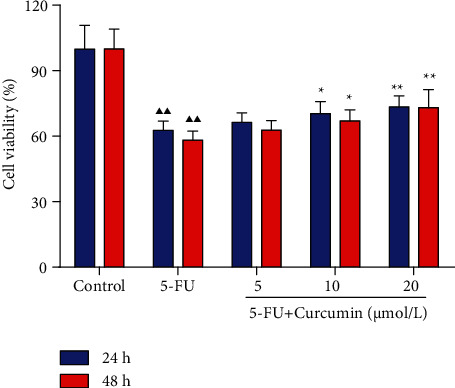
Effect of curcumin on the cell viability of the 5-FU-stimulated IEC-6 cells. IEC-6 cells were stimulated with 5-FU for 24 h and then further treated with curcumin at 5, 10, and 20 *μ*mol/L for 24 h or 48 h. CCK-8 assays were performed to detect the cell viability of each group cells. Data were expressed as mean ± standard error mean (SEM), ^▲▲^*p* value < 0.01 vs. control group; ^∗^*p* value < 0.05 vs. 5-FU group; ^∗∗^*p* value < 0.01 vs. 5-FU group. 5-FU: 5-fluorouracil.

As presented in Figure 1, 5-FU stimulation significantly inhibited the viability of intestinal epithelial IEC-6 cells compared to the untreated cells; the cell viability in the 5-FU group was reduced to approximately 60% after exposed to 5-FU for 24 h and 48 h. In the curcumin-treated groups, it could be observed that the coincubation with curcumin at 10 and 20 *μ*mol/L for 24 h and 48 h significantly improved the IEC-6 cell viability, compared with the 5-FU group. And there was no significant difference of the cell viability between 24 h and 48 h of treatment; thus, 24 h of incubation with curcumin was used for subsequent experiments.

### 3.2. Effect of Curcumin on the Apoptosis of the 5-FU-Stimulated IEC-6 Cells

**Figure 2:**
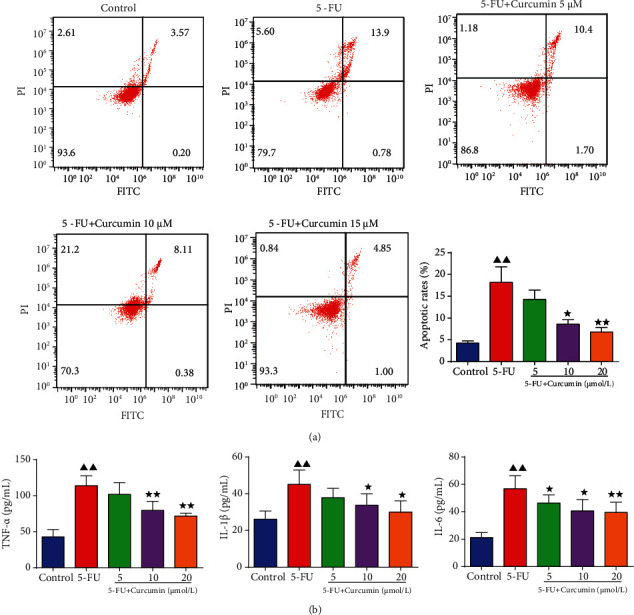
Effect of curcumin on the apoptosis and inflammation of the 5-FU-stimulated IEC-6 cells. IEC-6 cells were stimulated with 5-FU for 24 h and then further treated with curcumin at 5, 10, and 20 *μ*mol/L for 24 h. (a) Flow cytometry assay was performed to detect the cell apoptosis of each group cells. (b) The levels of TNF-*α*, IL-1*β*, and IL-6 were detected using ELISA kits. Data were expressed as mean ± standard error mean (SEM), ^▲▲^*p* value < 0.01 vs. control group; ^∗^*p* value < 0.05 vs. 5-FU group; ^∗∗^*p* value < 0.01 vs. 5-FU group. 5-FU: 5-fluorouracil.

After stimulated with 5-FU and treated with curcumin, the cell apoptosis was assessed. Relative results indicated that 5-FU stimulation significantly induced the apoptosis of IEC-6 cells, and the cell apoptotic rates were increased to approximately 18% in 5-FU group cells (Figure 2(a)). However, this proapoptosis effect on IEC-6 cells was reserved after curcumin treatment. It showed that in IEC-6 cells coincubated with curcumin, the cell apoptotic rates were decreased. And the cell apoptotic rates in curcumin 10 and 20 *μ*mol/L groups were below 10%, which also had statistical differences compared to the 5-FU group.

### 3.3. Effect of Curcumin on the Proinflammatory Cytokine Levels in the 5-FU-Stimulated IEC-6 Cells

The results in Figure 2(b) showed that the levels of TNF-*α*, IL-1*β*, and IL-6 were significantly increased in the 5-FU-stimulated IEC-6 cell group compared to the control group. In the curcumin-treated groups, it could be found that the levels of these proinflammatory cytokines were reduced, especially in curcumin 10 and 20 *μ*mol/L group cells.

### 3.4. Effect of Curcumin on the Cloning Ability and Migration of the 5-FU-Stimulated IEC-6 Cells

**Figure 3:**
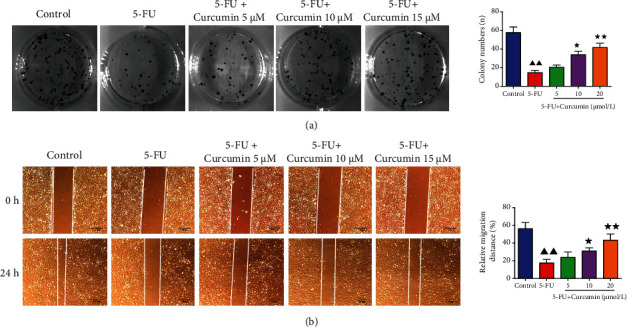
Effect of curcumin on the cloning ability and migration of the 5-FU-stimulated IEC-6 cells. IEC-6 cells were stimulated with 5-FU for 24 h and then further treated with curcumin at 5, 10, and 20 *μ*mol/L for 24 h. (a) Colony formation assay was performed to detect the cell colony ability of each group cells. (b) Wound healing assay was performed to detect the cell migration distance from 0 h to 24 h of each group cells. Data were expressed as mean ± standard error mean (SEM), ^▲▲^*p* value < 0.01 vs. control group; ^∗^*p* value < 0.05 vs. 5-FU group; ^∗∗^*p* value < 0.01 vs. 5-FU group. 5-FU: 5-fluorouracil.

**Figure 4:**
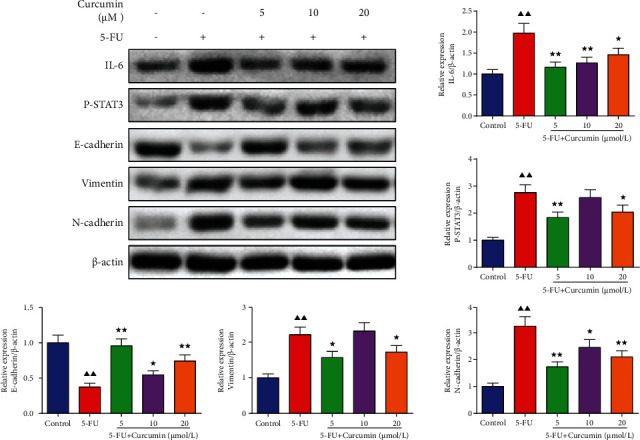
Effect of curcumin on the IL-6/STAT3 signaling in the 5-FU-stimulated IEC-6 cells. Western blotting assay was performed to detect the protein expression levels of E-cadherin, vimentin, N-cadherin, IL-6, and p-STAT3 in each group cells. Data were expressed as mean ± standard error mean (SEM), ^▲▲^*p* value < 0.01 vs. control group; ^∗^*p* value < 0.05 vs. 5-FU group; ^∗∗^*p* value <0.01 vs. 5-FU group. 5-FU: 5-fluorouracil.

Colony formation result in Figure 3(a) showed that the cloning ability of IEC-6 cells was reduced with the pretreatment of 5-FU, displayed by significantly decreased colony numbers in plate. In the curcumin treatment groups, it showed that the colony numbers were increased compared to the 5-FU group, especially in 10 and 20 *μ*mol/L concentrations. Cell migration assessment by wound healing assay (Figure 3(b)) showed that the cell migration viability inhibited by 5-FU was alleviated by curcumin treatment, from 0 h to 24 h. The relative cell migration distance in the curcumin groups was increased compared to the 5-FU group, but the control group cells still had the longest migration distance. Western blotting also detected the migration-related factors, including E-cadherin, vimentin, and N-cadherin. Results in Figure 4 showed that their relative protein levels were changed after 5-FU stimulation, and E-cadherin was decreased, while vimentin and N-cadherin were increased significantly, and after treated with curcumin, their changed expression levels were recovered in a certain extent.

### 3.5. Effect of Curcumin on the IL-6/STAT3 Signaling in the 5-FU-Stimulated IEC-6 Cells

As previous study indicated that curcumin has regulation effect on IL-6/STAT3 signaling, we also detected the protein expression levels of genes in IL-6/STAT3 signaling pathway. As shown in Figure 4, it could be found that 5-FU exposure activated the expression levels of IL-6 and p-STAT3 in IEC-6 cells. And after curcumin treatment, the expression levels of IL-6 and p-STAT3 were reduced in 5, 10, and 20 *μ*mol/L group cells, indicating the inhibition effect of curcumin on the IL-6/STAT3 signaling in 5-FU-stimulated IEC-6 cells.

### 3.6. Identification of DEGs in Curcumin-Treated IEC-6 Cells

**Figure 5:**
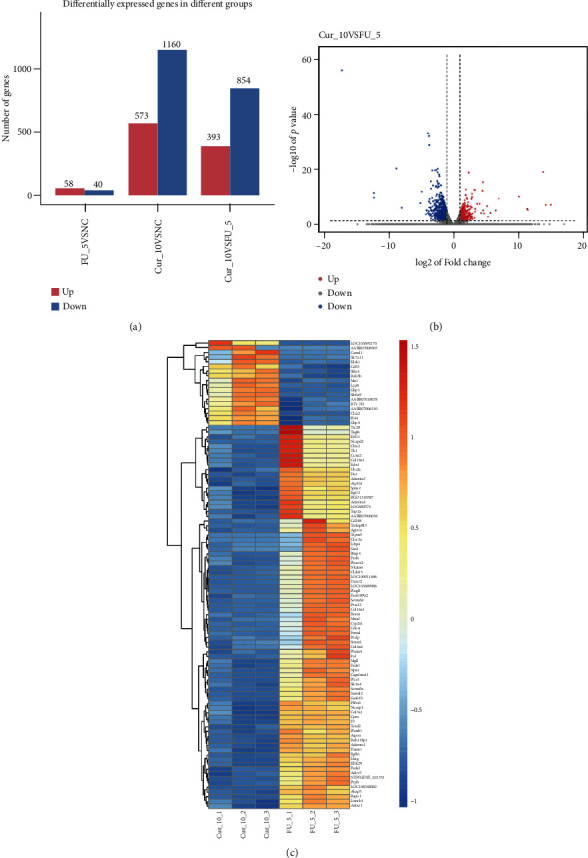
RNA-sequencing and identification of DEGs in curcumin-treated IEC-6 cells. (a) Identification of DEGs between the tested groups according to the thresholds of *p* < 0.05 and ∣logFC | >1. (b) Volcano plot of the identified DEGs between the 5-FU and 5-FU + curcumin groups. Blue plot represented downregulated DEGs; red plot represented upregulated DEGs. (c) Cluster analysis of the top 100 DEGs. 5-FU: 5-fluorouracil; Cur: curcumin; DEGs: differentially expressed genes.

The IEC-6 cells from the control, 5-FU, and 5-FU + curcumin (10 *μ*mol/L) groups were further collected for RNA-sequencing analysis to identify curcumin-associated genes. According to the thresholds of *p* < 0.05 and ∣logFC | >1, a total of 1247 DEGs were selected between the 5-FU and 5-FU + curcumin groups, including 393 upregulated DEGs and 854 downregulated DEGs (Figure 5(a)). The volcano plot of the identified DEGs is presented in Figure 5(b). Cluster analysis of the top 100 DEGs in Figure 5(c) showed that these DEGs had significant clustering between the 5-FU and curcumin group cell samples.

### 3.7. PPI Network Analysis of the DEGs

**Figure 6:**
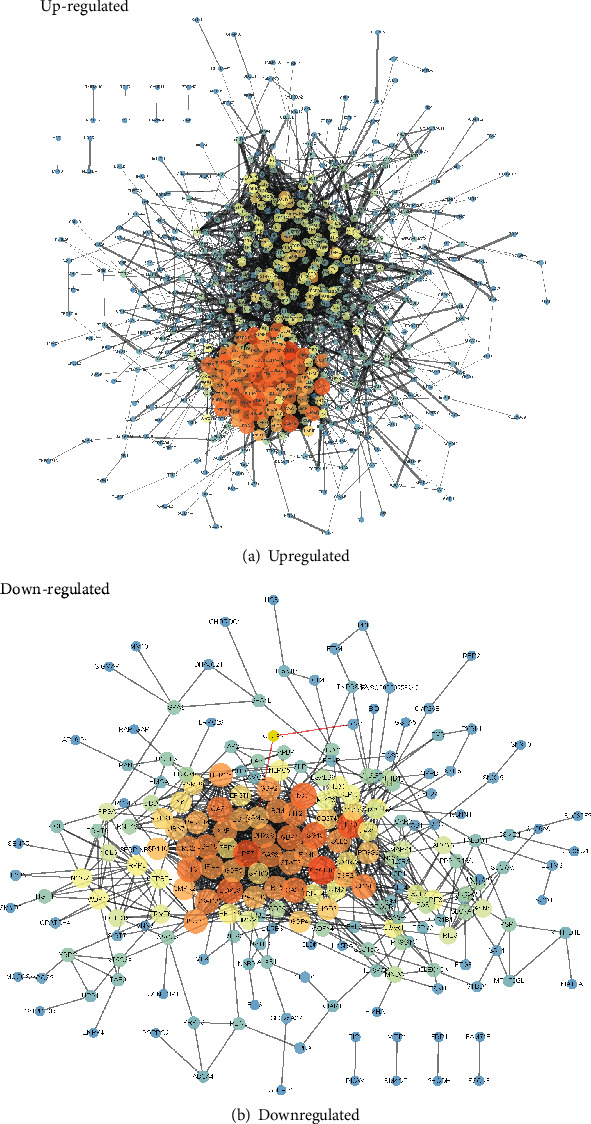
PPI network analysis of the DEGs: (a) PPI network of the upregulated DEGs; (b) PPI network of the downregulated DEGs.

PPI networks of the upregulated and downregulated DEGs were constructed by the STRING database (Figure 6). A total of 1059 edges and 217 nodes were included in the upregulated DEGs' PPI network (Figure 6(a)). The CXCL10 (degree = 54), IL10 (degree = 51), IRF7 (degree = 49), ISG15 (degree = 43), and MX1 (degree = 43) were selected as key upregulated DEGs in the network because of their high node degree. For the downregulated DEGs' PPI network, there were 675 DEGs mapped into the network, forming 675 nodes and 5112 edges in the network (Figure 6(b)). Also, CDK1 (degree = 111), CCNB1 (degree = 98), IL6 (degree = 98), and CDC20 (degree = 96) were selected as key downregulated DEGs in the network because of their high node degree.

### 3.8. Functional Enrichment Analysis of the DEGs

**Figure 7:**
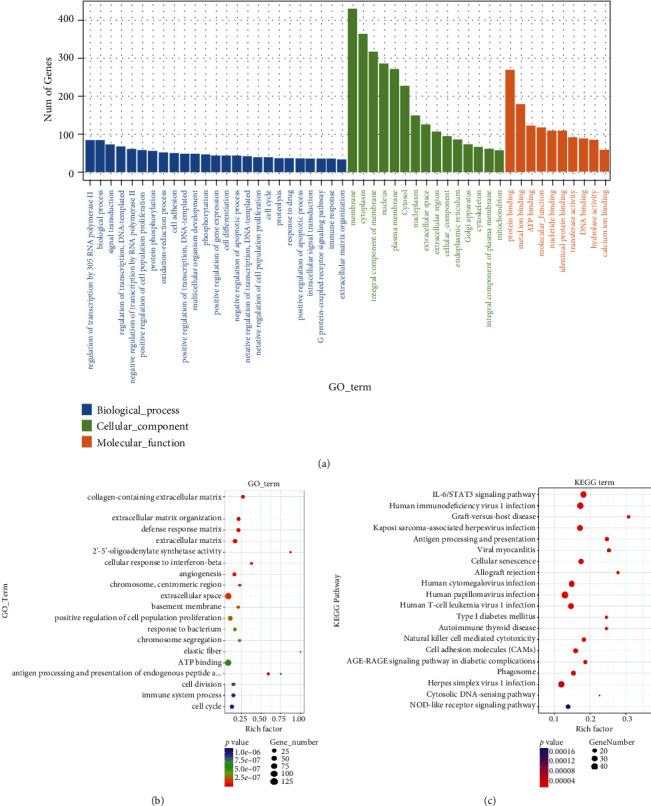
Functional enrichment analysis of the DEGs: (a, b) GO functional enrichment analysis of the DEGs; (c) KEGG pathway enrichment analysis of the DEGs. GO: Gene Ontology; KEGG: Kyoto Encyclopedia of Genes and Genomes.

GO functional enrichment analysis of the DEGs showed that these DEGs were significantly enriched in biological process terms of regulation of transcription by RNA polymerase II, positive regulation of cell population proliferation, protein phosphorylation, and cell adhesion; in molecular function terms of protein binding, metal ion binding, ATP binding, nucleotide binding, and identical protein binding; and in cellular component terms of cytoplasm, cytosol, extracellular space, extracellular region, endoplasmic reticulum, etc. (Figures 7(a) and 7(b)). KEGG pathway enrichment analysis of the DEGs showed that these DEGs were enriched in pathways including IL-6/STAT3 signaling pathway, pathways in cancer, human papillomavirus infection, herpes simplex virus 1 infection, human immunodeficiency virus 1 infection, human cytomegalovirus, and human T-cell leukemia virus 1 infection (Figure 7(c)).

### 3.9. Validation of IL-6 and STAT3 Levels in Transfected IEC-6 Cells

**Figure 8:**
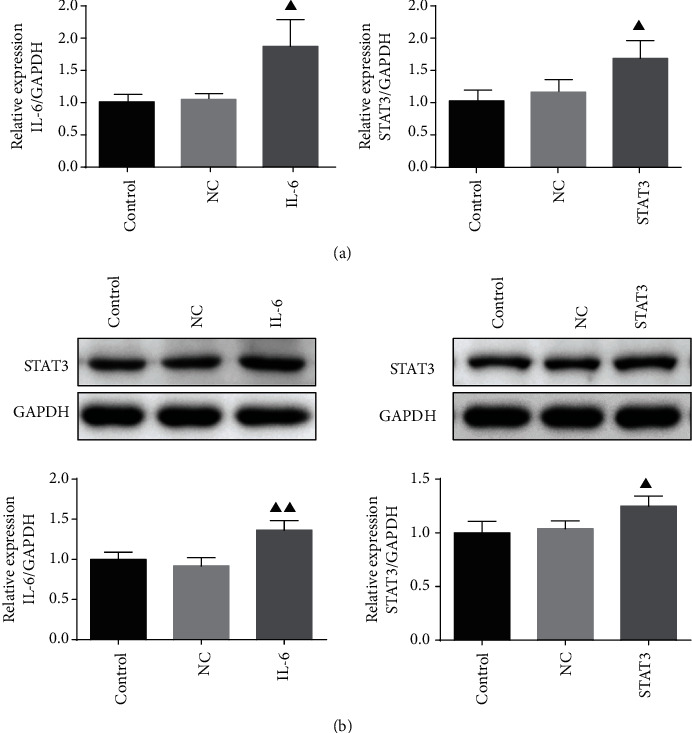
Validation of IL-6 and STAT3 levels in transfected IEC-6 cells. IEC-6 cells were transfected with STAT3 overexpressed plasmid and IL-6 overexpressed plasmid, respectively; the expression of IL-6 and STAT3 in transfected IEC-6 cells was validated by qRT-PCR analysis (a) and western blot assay (b). Data were expressed as mean ± standard error mean (SEM), ^▲^*p* value < 0.05 vs. control group.

After IEC-6 cells were transfected with STAT3 overexpressed plasmid or IL-6 overexpressed plasmid, respectively, the expressions of IL-6 and STAT3 in transfected IEC-6 cells were validated. As shown in Figures 8(a) and 8(b), the mRNA and protein expressions of IL-6 and STAT3 were all significantly higher in transfected cells compared to the control group or vector NC group cells.

### 3.10. Overexpression of IL-6 and STAT3 Reserved the Effect of Curcumin on 5-FU-Stimulated IEC-6 Cells

**Figure 9:**
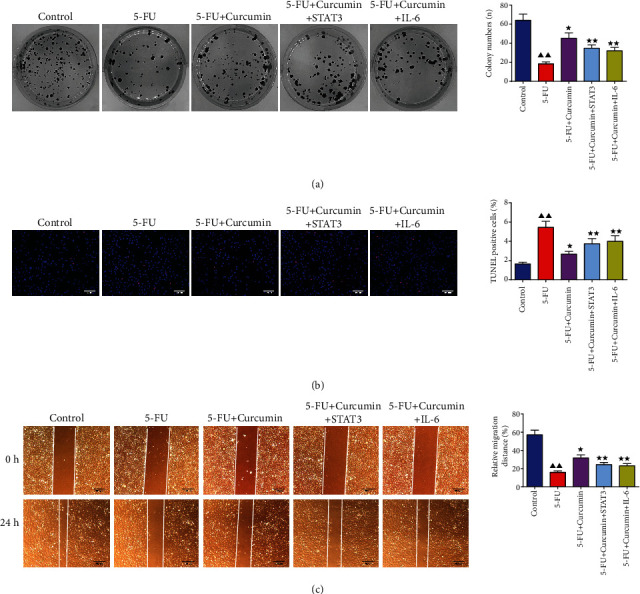
Overexpression of IL-6 and STAT3 reserved the effect of curcumin on 5-FU-stimulated IEC-6 cells. (a) Colony formation assay. (b) TUNEL staining. (c) Wound healing assay. Data were expressed as mean ± standard error mean (SEM), ^▲▲^*p* value < 0.01 vs. control group; ^∗^*p* value < 0.05 vs. 5-FU group; ^∗∗^*p* value < 0.01 vs. 5-FU group. 5-FU: 5-fluorouracil.

Based on the above results, we assumed that curcumin displayed the protective effects on IEC-6 cells against 5-FU by IL-6/STAT3 signaling. Then, the influence of IL-6/STAT3 signaling on curcumin in 5-FU-stimulated IEC-6 cells was evaluated. As displayed in Figure 9, the results showed that compared to the 5-FU + curcumin group cells, the protective effects of curcumin on 5-FU-stimuted IEC-6 cells were overturned in IL-6 and STAT3 overexpression transfected cells. Compared to those in the 5-FU + curcumin group cells, overexpression of IL-6 and STAT3 inhibited the colony numbers, increased TUNEL positive cells, and decreased the migration distance. Taken together, these data manifested that curcumin protects against 5-FU-induced intestinal epithelial cell damage via downregulating of IL-6/STAT3 signaling.

## 4. Discussion

5-FU, served as an antimetabolite chemotherapy drug, displays its effect through interrupting DNA synthesis, leading to cell death by proapoptosis. However, the clinical use of 5-FU for the treatment of cancers, including colorectal cancer, is also accompanied with dose-dependent toxicities, such as IM [2, 3]. This adverse effect negatively impacts on therapeutic outcomes and prognosis of cancer patients. In present study, in an *in vitro* model of IM, we demonstrated that curcumin efficiently attenuated cell damage of intestinal epithelial cells exposed to 5-FU. 5-FU caused inhibition on IEC-6 cell viability, colony formation and migration ability, and induced cell apoptosis, which were restored by curcumin treatment.

As a natural product with a wide range properties, curcumin also displays potential on the gastrointestinal system disorders. It is reported to have the ability to suppress the inflammation and improve intestinal barrier function in inflammatory bowel disease [16, 17]. This therapeutic effect may contribute to its anti-inflammatory, antimicrobial, immunomodulatory properties, the modulation of signaling mediators, and tight junctions. Based on abundant preclinical findings, clinical trials assessed the therapy efficacy of curcumin in multiple types of diseases also yielded beneficial data [18]. Moreover, poor bioavailability of curcumin is overcame by in combination with other compounds or as a nanocarrier formulation [19, 20]. In cancer treatment, curcumin is capable of reserving the toxicity induced by chemotherapy drugs, including IM. Sakai et al. reported that curcumin administered can prevent the symptoms of diarrhea induced by 5-FU in mice [21]. In this study, in IEC-6 cells stimulated with 5-FU, our results showed that curcumin efficiently attenuated 5-FU-induced intestinal epithelial cell damage, attenuated 5-FU-induced inhibition on cell viability, and displayed antiapoptosis effect on IEC-6 cells. This antiapoptosis effect on IEC-6 cells was also reported in Ouyang et al.'s study [22]. In the irinotecan-induced intestinal mucosal injury model in nude mice, curcumin was found to attenuate the development of diarrhea and intestinal mucosa damage effectively and also improved cell morphology, inhibited apoptosis, and suppressed oxidative stress in irinotecan-treated IEC-6 cells.

When we further investigated the impact of 5-FU on IEC-6 cells and protective effect of curcumin, relative experiments showed that 5-FU stimulation induced inflammation and damage, while curcumin displayed its protective effect against cell damage through inflammation-related mediators and signaling. Inflammation is a major event in IM; the pathogenesis of IM involves in the interfere of inflammatory mediators, which are mainly over produced by 5-FU or other drug stimulation [2, 23]. In Dark Agouti rat injected with 5-FU, there is a markedly increase of NF-*κ*B, TNF-*α*, IL-1*β*, and IL-6 expression in oral mucosa, jejunum, and colon tissues [10]. A study investigating the impact of 5-FU chemotherapy on gut inflammation showed that in AOM/DSS-induced colorectal cancer model mouse treated with 5FU, significantly elevated expression of intestinal inflammatory genes was found, including TNF-*α*, MCP-1, NOS2, IL6, IL1-*β*, and FOXP3 [24]. In this study, ELISA assay and Western blotting results showed that in 5-FU-suppressed IEC-6 cells, the expression levels of TNF-*α*, IL1-*β*, IL-6, and STAT3 were increased, indicated the presence of inflammation in intestinal epithelial cells with 5-FU, which contributed to the development of IM. In addition, curcumin treatment suppressed the expression of these mediators.

More importantly, RNA-sequencing analysis in this study identified 393 upregulated DEGs and 854 downregulated DEGs in curcumin-treated IEC-6 cells. KEGG pathway analysis also found that these DEGs were enriched in IL-6/STAT3 signaling. Further experimental validation of IEC-6 cells transfected with IL-6 overexpressed plasmid or STAT3 overexpressed plasmid showed that curcumin protected against intestinal epithelial cell damage via the downregulation of IL-6/STAT3 signaling. IL-6 is a critical mediator stimulated by a variety of cells, including T lymphocytes, epithelial cells, monocytes, macrophages, and tumor cells. Elevated levels of IL-6 are often observed in chemotherapy-induced IM, which closely correlate with intestinal pathophysiological condition and gut homeostasis, including inflammation promoting [25, 26]. And studies focused on reducing symptoms of various intestinal diseases, such as IM, are always accompanied by lower IL-6 level [27–29]. STAT3 is a molecular in STAT family with crucial effects on cell proliferation, differentiation, migration, and angiogenesis in cancer progression; persistent STAT3 activation is found to promote chronic inflammation, which increases susceptibility of healthy cells to carcinogenesis [30, 31]. When the intestinal mucosa is stimulated, elevated IL-6 can activate the phosphorylation of STAT3 and response to NF-*κ*B, a central mediator of intestinal inflammation, to promote the injury of inflammatory factors to intestinal epithelial cells [32]. Several genome-wide studies on intestinal disorder patients identified variants of STAT3 as a risk factor for diseases development, and the activated expression of STAT3 in intestinal cells, including intestinal epithelial cells, was also detected in inflamed intestinal regions [33, 34]. Therefore, IL-6/STAT3 signaling pathway is important for intestinal homeostasis maintenance, and regulation of this signaling is an approach in treatment of intestinal disorders.

Previous studies also proved the regulation ability of curcumin on IL-6/STAT3 signaling pathway. Curcumin is reported as an inhibitor of p-STAT3, to abrogate the IL-6-induced phosphorylation of STAT3, and inhibits the proliferation and survival of multiple myeloma cells [35]. In psoriasis mice induced by imiquimod, curcumin could improve the pathological injuries of the mouse skin, reduced the expressions of IL-6, p-STAT3, and its downstream proteins. In intestinal disorders including IM, curcumin exerts its anti-inflammatory effects not only by inhibiting NF-*κ*B activation but also contributes to its ability to regulate IL-6/STAT3 signaling. Curcumin is reported to exert therapeutic effects in experimental colitis induced by dextran sulfate sodium (DSS) through blocking STAT3 signaling pathway [36]. Curcumin can alleviate the severity of colitis by suppressing the expression of STAT3, NF-*κ*B, COX-2, and iNOS [37]. Except for the anti-inflammation effect by curcumin in IM, the protective role of curcumin also attributed to maintain intestinal mucosal barrier function and intestinal microbe homeostasis.

In conclusion, the present study used a chemotherapy drug, 5-FU, to induce a model of IM in intestinal epithelial cells; the results showed that curcumin efficiently attenuated 5-FU-induced IEC-6 cell damage and inflammation, attenuated the 5-FU-induced inhibition on cell viability, and displayed antiapoptosis effect on IEC-6 cells. Further RNA-sequencing analysis and experiments also found that curcumin displayed its protective effect against 5-FU-induced IM in intestinal epithelial cells by the inhibition of IL-6/STAT3 signaling pathway. Taken together, these findings suggested that curcumin may be provided as an adjunctive agent in alleviation of chemotherapy-induced IM.

## Data Availability

The data used to support the findings of this study are included in this manuscript.
